# Bridging the industrial data gap: Top-down approach from national statistics to site-level energy consumption data

**DOI:** 10.1016/j.dib.2025.111365

**Published:** 2025-02-03

**Authors:** Enrico Bernelli Zazzera, Matteo Giacomo Prina, Riccardo Marchetti, Steffi Misconel, Giampaolo Manzolini, Wolfram Sparber

**Affiliations:** a EURAC Research, Institute for Renewable Energy, Viale Druso 1, I-39100 Bolzano, Italy; b Dipartimento di energia, Politecnico di Milano, Via Lambruschini, 4, 20156 Milano, MI, Italy

**Keywords:** Industrial energy consumption, Energy balance disaggregation, Industrial emissions, Hard-to-abate sectors, Energy system modelling

## Abstract

Detailed data on hard-to-abate industrial sectors is crucial for developing targeted decarbonization measures in energy system modeling, yet such information is rarely available through open sources. This paper presents a top-down methodology to estimate detailed industrial site-level energy and emissions databases by integrating and expanding publicly available data. The methodology addresses three key challenges: (1) the disaggregation of national energy consumption data to site level, (2) the categorization of process heat by four temperature ranges (<100 °C, 100 °C-500 °C, 500 °C-1000 °C, and >1000 °C) and direct use of electricity, and (3) the integration of process emissions from feedstock use in hard-to-abate industrial sectors. The approach is demonstrated through application to the Italian industrial sector for the year 2022, resulting in a database that documents site-specific consumption across seven energy sources: solid fossil fuels, manufactured gases, oil and petroleum products, natural gas, biofuels, non-renewable wastes, naphtha and electricity. The method can be replicated for other European countries, providing researchers and policymakers with a standardized approach to create detailed industrial energy databases. Results show that the chemical and petrochemical sector dominates the industrial energy landscape of Italy, followed by iron and steel, non-metallic minerals, and paper and pulp. The geographical distribution reveals a concentration of major industrial facilities in northern Italy, with notable exceptions including significant steel production in Taranto (south) and petrochemical complexes in Sicily and Sardinia.

Specifications Table

**Table:** 

Subject	Renewable Energy, Sustainability and the Environment
Specific subject area	Industrial energy consumption, Process engineering, Carbon emissions
Type of data	Table, Excel file
Data collection	Data were downloaded in excel of csv format from different open sources.
Data source location	European Union Emissions Trading System (EU ETS), Statistics | Eurostat, JRC-IDEES-2021, ATECO 2007 industrial classification system, Italian municipalities geographical database.
Data accessibility	Repository name: Italian Industrial Site-Level Energy and Emissions Database 2022: A Top-down Disaggregation Methodology Data identification number: 10.5281/zenodo.14417812 Direct URL to data: https://zenodo.org/records/14417813
Related research article	None

## 1 Value of the Data

• The database provides unprecedented granularity in industrial energy consumption patterns at site level, enabling researchers and policymakers to develop targeted decarbonization strategies for specific industrial facilities and sectors.
• Energy system modelers can utilize this detailed breakdown of final energy consumption by temperature ranges (<100 °C, 100 °C-500 °C, 500 °C-1000 °C, >1000 °C), direct electricity use and feedstock to identify and assess specific decarbonization options.
• The site-level geographical information enables the assessment of: regional industrial clusters and potential synergies, infrastructure requirements for different decarbonization pathways, renewable energy resource availability for specific industrial sites, district heating network potential, local grid capacity needs for electrification.
• The methodology based on open data and basic assumptions can be replicated for other European countries, facilitating: cross-country comparisons of industrial energy use patterns, identification of best practices and technology transfer opportunities, assessment of pan-European industrial decarbonization strategies.

## 2 Background

The decarbonization of the industrial sector represents one of the most challenging aspects of the energy transition. A detailed understanding of energy consumption patterns and emissions across different industrial sectors is crucial for identifying and implementing effective decarbonization strategies for several key reasons:

• Process-specific technology solutions: Different industrial processes require energy at varying temperature levels and in different forms (thermal, electrical, chemical). Understanding these specific requirements is essential for identifying suitable low-carbon alternatives. For instance, low-temperature processes might be electrified using heat pumps, while high-temperature processes might require hydrogen or other emerging technologies.
• Infrastructure planning: Site-level energy consumption data enables better planning of future infrastructure needs, such as electricity grid reinforcement for electrification, hydrogen pipeline networks, or CO_2_ transport infrastructure for carbon capture and storage.
• Regional strategy development: Detailed geographical information about industrial energy use helps identify potential industrial clusters where shared infrastructure and symbiotic relationships between facilities could reduce decarbonization costs.
• Investment prioritization: Understanding which sectors and sites are responsible for the largest emissions helps policymakers and industry stakeholders prioritize investments and interventions for maximum impact.

However, there is a significant gap in the availability of comprehensive industrial energy databases that provide detailed breakdowns of energy consumption by temperature ranges, fuel types, and feedstock uses across different industrial sectors.

Table 1 shows currently available databases and their specific limitations showing that existing databases offer partial views of industrial energy use and emissions.

**Table 1:** Available databases on energy consumption and emissions of the industry sector.

Name	Last update	Data available	Limitations
EU-ETS [1]	Updated every year	CO₂ emissions data of major European industrial sites, with details on identifiers and location	Consider just the largest industrial sites. Lacks industry sector attributes.
Hotmaps [2]	2014	CO₂ emissions, production, fuel demand and excess heat of some of the largest industrial sites in Europe.	Outdated and incomplete data.
National TSO (Terna) [3]	Updated every year	Electricity consumption data for industrial sectors detailed by province and region.	No information on specific industrial sites and fuel consumption
Energy Balances (EUROSTAT) [4]	Updated every year	Energy consumption of industrial sectors, by energy source.	No information on specific industrial sites
Diversity index of final energy consumption by sector (EUROSTAT) [5] JRC Integrated Database of the European Energy System (JRC-IDEES) [6]	Updated every year 2021	Energy sources utilized within an industrial sector. Disaggregated energy-economy-emissions data for each Member State of the European Union, covering all sectors of the energy system and specific industrial processes for the 2000–2021 period: industry, buildings, transport, and power generation.	Lacks information on the specific usage of energy sources within industrial sectors. No information on specific industrial sites. Some industrial sectors are not included in the database.

## 3 Data Description

The resulting database provides a comprehensive mapping of energy consumption and emissions for Italian industrial sites in 2022. The database structure includes multiple levels of information for each facility: geographical data (coordinates, region, city), industrial sector classification (both detailed ATECO class and macro-sector category), verified emissions from EU-ETS, and calculated energy consumption patterns. The energy consumption is detailed across eight energy carriers (solid fossil fuels, manufactured gases, oil and petroleum products, natural gas, biofuels, non-renewable wastes, naphtha and electricity) plus feedstock use where applicable. Additionally, the thermal energy demand is categorized into four temperature ranges, providing crucial insights for technology replacement and process optimization strategies.

**Fig. 1:**
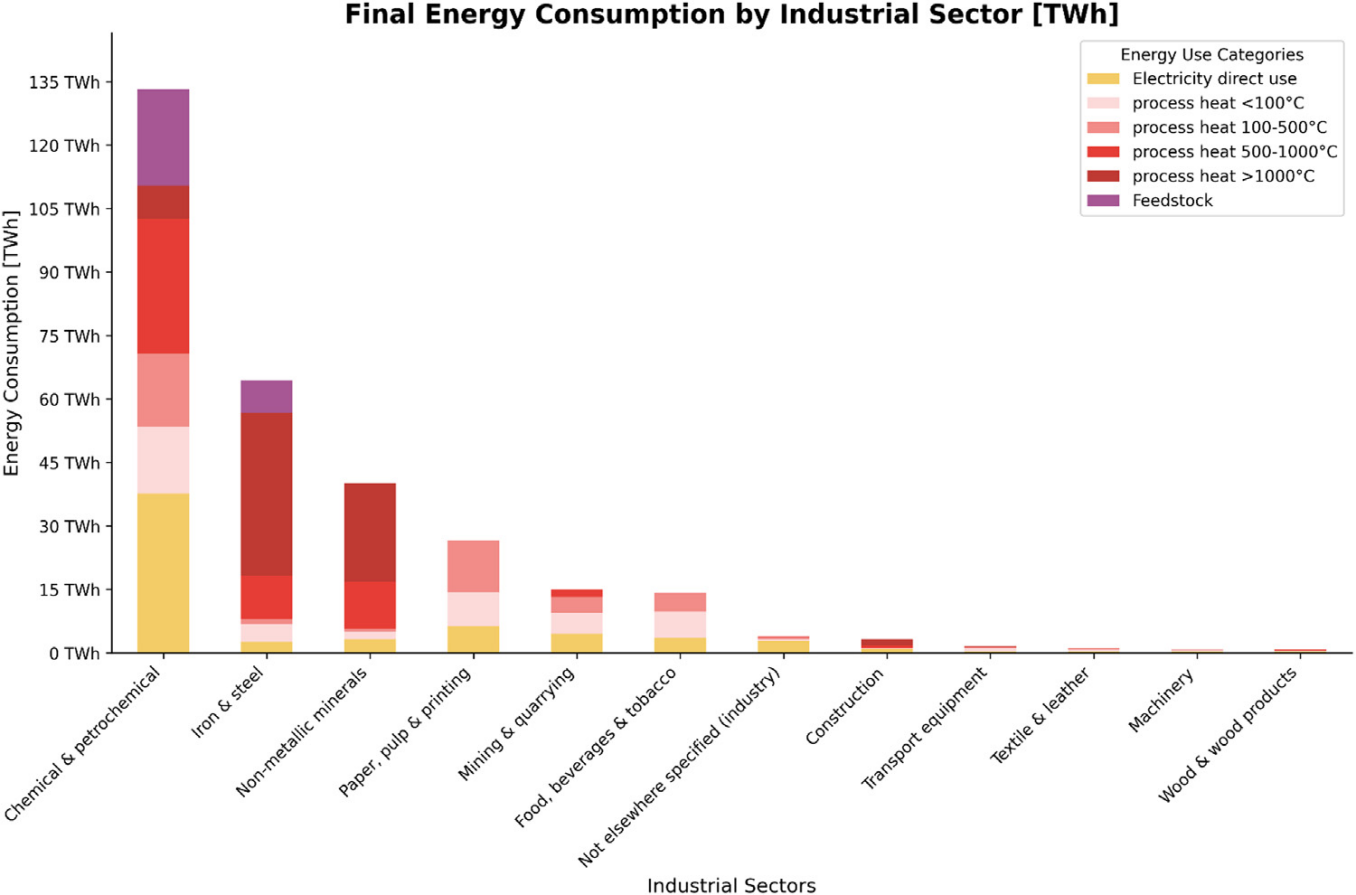
Final energy consumption by industrial sector in Italy, 2022.

Fig. 1 illustrates the final energy consumption across Italian industrial sectors in 2022. Final energy consumption refers to the total energy consumed by end users, such as households, industries, and businesses, for their energy needs. It represents the energy that reaches the final user's door after all conversion and transmission losses, and excludes the energy used by the energy sector itself, including for transformation and distribution. In the industrial context, final energy consumption includes all energy carriers (such as electricity, natural gas, coal, oil products) directly used in manufacturing processes, heating, cooling, lighting, and powering machinery. This differs from primary energy consumption, which includes the energy losses that occur during the conversion of primary sources (like coal or crude oil) into the final energy carriers that reach industrial consumers. The four main energy-intensive sectors are Chemical & Petrochemical, Iron & Steel, Non-metallic minerals, and Paper, pulp & printing. Notably, high-temperature processes (>500 °C) dominate energy consumption in Iron & Steel and Non-metallic minerals sectors, which is primarily due to steel production processes and clinker production in cement manufacturing, respectively.

**Fig. 2:**
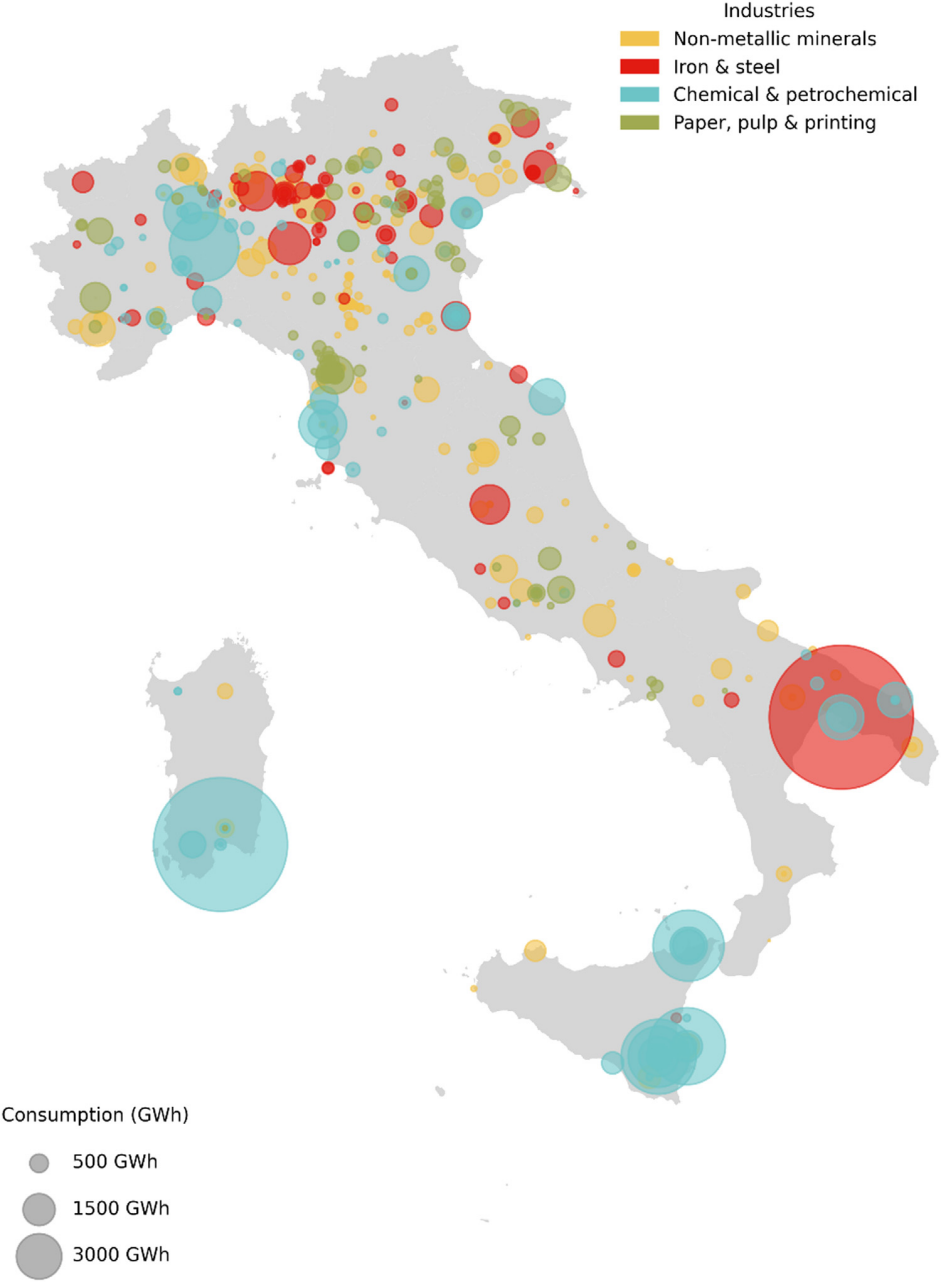
Final energy consumption at industrial site-level for the 4 main energy-intensive industrial sectors in Italy, 2022.

Fig. 2 presents a geographical distribution of site-level energy consumption for Italyʼs four main energy-intensive industries in 2022. The map reveals significant regional concentrations, with the largest iron and steel facility located in Taranto (southeastern Italy), major chemical and petrochemical clusters in Sardinia and Sicily, and numerous non-metallic mineral facilities (primarily cement plants) distributed across the mainland. The size of the circles represents the magnitude of energy consumption, highlighting the varying scales of industrial operations across different regions.

The developed dataset provides detailed granularity in mapping Italyʼs industrial energy landscape, offering several key advantages for energy system modeling and scenario analysis. By combining EU-ETS emissions data, EUROSTAT energy balances, and detailed process heat temperature requirements, this database enables a more nuanced understanding of industrial energy demands across different temperature ranges and fuel types at the site level.

This granular information is particularly valuable for energy system modeling as it allows for:

1. Precise assessment of technology substitution potential (e.g., evaluating where heat pumps could replace fossil fuel-based heating for low-temperature processes, or where hydrogen might be viable for high-temperature applications)
2. Regional analysis of industrial clusters and potential synergies (such as waste heat utilization or shared infrastructure development)
3. Detailed evaluation of sector-specific decarbonization pathways, considering both energy and feedstock-related emissions

Furthermore, the methodology's replicability across other European countries makes it a valuable tool for coordinated industrial decarbonization planning at both national and European levels. The combination of geographical distribution, temperature requirements, and energy carrier specificity provides essential inputs for developing targeted and cost-effective transition strategies that account for local industrial characteristics and resource availability.

## 4 Experimental Design, Materials and Methods

**Fig. 3:**
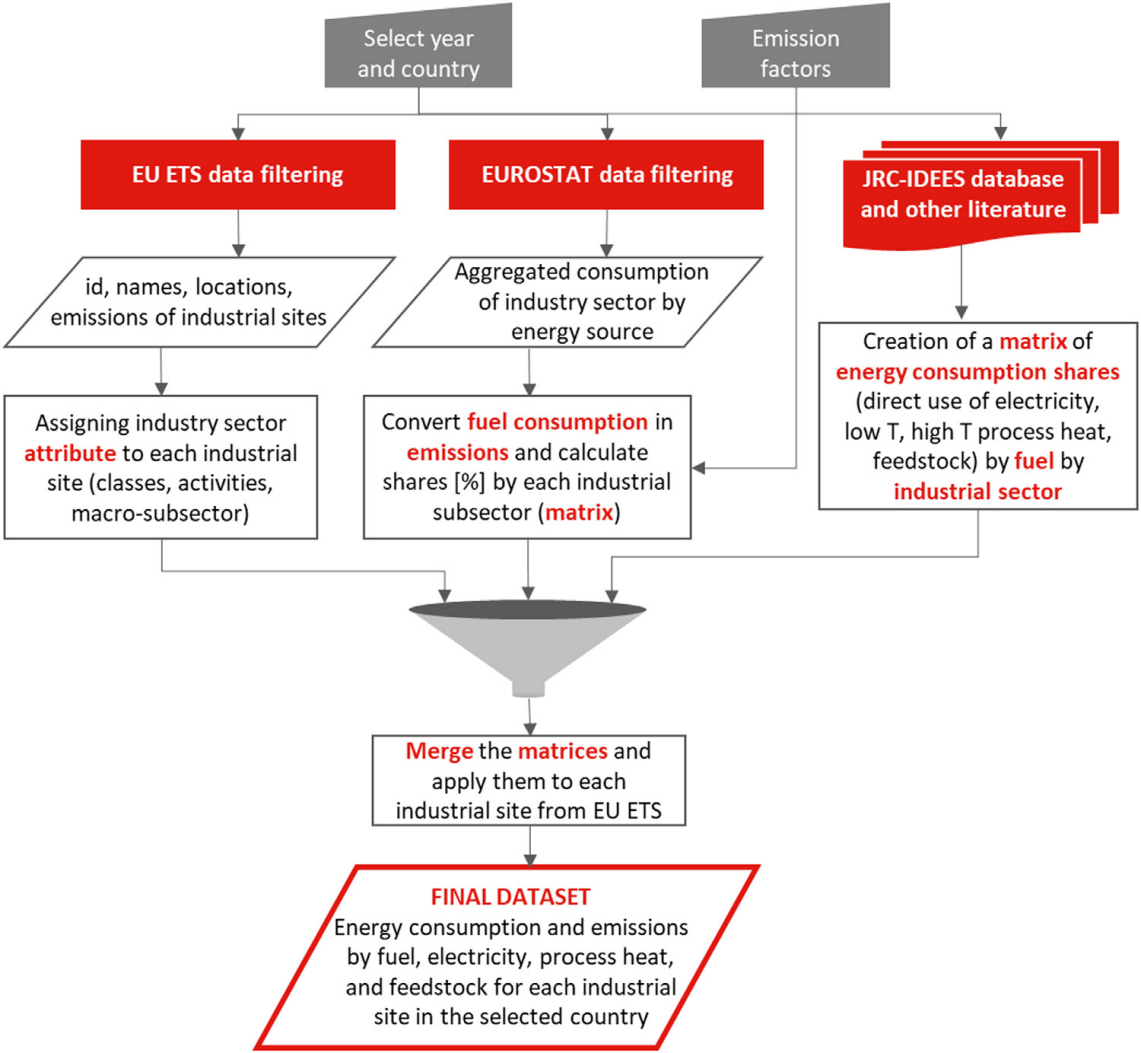
Flowchart of the methodology for creating a site-level industrial energy consumption and emissions database.

This section describes the methodology developed to create a comprehensive database of energy intensive industrial site-level energy consumption and emissions for Italy. The approach integrates multiple data sources, primarily the European Union Emissions Trading System (EU-ETS) database, EUROSTAT Energy Balance statistics, and the JRC-IDEES, supplemented with the ATECO 2007 industrial classification system and Italian geographical data. The methodology follows three main steps: (1) compilation and enhancement of site-level emissions data from EU-ETS, (2) transformation of sectoral energy consumption data from EUROSTAT into site-specific energy distributions, and (3) definition of electricity consumption for direct use and for process heat generation, and share of process emissions from feedstock use for the hard-to-abate sectors (Iron & Steel, Chemical & Petrochemical, Non-metallic minerals) from JRC-IDEES (4) categorization of energy use across temperature ranges (<100 °C, 100 °C-500 °C, 500 °C-1000 °C, and >1000 °C). As shown in Fig. 3 the methodology follows three parallel streams that converge to create the final database.

The four streams are the following:

(1) EU-ETS stream: The European Emission Trading System database [1] contains information about the emissions of the energy-intensive industrial sites across Europe. Different criteria define the industrial sites to be listed within this database such as a production capacity exceeding 2.5 tonnes per hour for iron and steel industry or a production capacity exceeding 20 tonnes per day for paper sector [7]. From the original dataset, the following screening process has been followed: i) Only the record with the country code IT (Italy) and the year 2022 was selected Ii) Only active installations with valid PERMIT_ENTRY_DATE and current PERMIT_IDENTIFIER were included in the analysis Iii) Sites were screened based on their VERIFIED_EMISSIONS_2022 values, with installations reporting zero or missing emissions being excluded from the analysis. The following variables were extracted from this dataset: INSTALLATION_IDENTIFIER, INSTALLATION_NAME, PERMIT_ENTRY_DATE, ENTRY YEAR, CITY, ADDRESS1, MAIN_ACTIVITY_TYPE_CODE, PERMIT_IDENTIFIER, LAST_NAME, and VERIFIED_EMISSIONS_2022. Subsequently, two additional attributes, namely CLASSES and MACRO_CATEGORIES, were incorporated into the database to facilitate the classification of entities according to their respective industry belonging. The initial attribute is more specific and serves to indicate the particular activity that is undertaken at the industrial site, based on the ATECO 2007 classification system [8]. The second attribute is more generic and serves to indicate the industry sector to which the entity in question belongs. To facilitate the work, the definition of the industrial sector was based on the classification present in the EUROSTAT Energy Balance statistics [4]. Finally, in order to improve the geolocalisation of the industrial sites, information on the coordinates and the region to which they belong has been added. This was done by merging the data with a database of Italian municipalities [9], using the postal code as a key. To avoid double counting, the EU ETS focuses on direct fuel combustion and does not capture electricity consumption, since these emissions are accounted for at the power generation stage. Thus, it was essential to exclude all industrial facilities classified as Power Generation and Distribution, as the objective is to concentrate exclusively on the energy-intensive industrial domain.
(2) The energy balance statistics for industrial subsectors provided by EUROSTAT was utilized to enable the conversion from ETS emissions to final energy consumption [4]. The first step involved converting the data from energy source consumption [MWh] to the corresponding emissions [tCO_2_], using a set of emissions factors [[10], [11], [12]]. Having obtained the emissions data related to different energy sources and feedstock use (see stream 3) for each industrial sector, it is now possible to translate this data into percentage terms in order to define an “emission mix” that describes the distribution of emission sources for each industry, as illustrated in Table 2. It is noteworthy to mention that has been decided to not consider the term “heat” from the statistics, as it is presumed to be associated with the heating sector rather than the industrial one.

**Table 2:** Shares of fuel emissions by industrial sector in Italy (2022).

Industry sector	Solid fossil fuels	Manufactured gases	Oil and petroleum products	Natural gas	Renewables and biofuels	Non-renewable waste	Feedstock emissions
**Iron & Steel**	13.30 %	3.30 %	8.70 %	51.81 %	0.00 %	0.00 %	22.90 %
**Chemical& Petrochemical**	0.00 %	0.00 %	48.46 %	23.01 %	0.61 %	3.41 %	24.52 %
**Non-metallic minerals**	0.34 %	0.00 %	23.27 %	20.22 %	2.73 %	2.95 %	50.50 %
**Transport equipment**	0.00 %	0.00 %	0.00 %	100.00 %	0.00 %	0.00 %	0.00 %
**Machinery**	0.00 %	0.00 %	26.51 %	73.23 %	0.26 %	0.00 %	0.00 %
**Mining & quarrying**	0.00 %	0.00 %	12.95 %	87.05 %	0.01 %	0.00 %	0.00 %
**Food, beverages & tobacco**	0.00 %	0.00 %	12.35 %	83.93 %	3.73 %	0.00 %	0.00 %
**Paper, pulp & printing**	0.00 %	0.00 %	18.53 %	80.97 %	0.50 %	0.00 %	0.00 %
**Wood & wood products**	0.00 %	0.00 %	0.00 %	24.05 %	72.99 %	2.97 %	0.00 %
**Construction**	0.00 %	0.00 %	1.63 %	97.76 %	0.60 %	0.00 %	0.00 %
**Textile & leather**	0.00 %	0.00 %	9.38 %	89.02 %	1.61 %	0.00 %	0.00 %
**Not elsewhere specified**	0.00 %	0.00 %	5.47 %	70.74 %	16.22 %	7.56 %	0.00 %
(3) A significant challenge was to incorporate all emissions that are not directly attributable to the combustion of fossil fuels but are instead the result of chemical reactions occurring within production processes, and thus to the utilization of specific materials as feedstock. Such emissions are only present in so-called hard-to-abate sectors, as the presence of these 'process' emissions makes decarbonization a significant challenge. The sectors in question are chemicals and petrochemicals, cement, and iron and steel. This issue was addressed through the utilization of the JRC-IDEES, which provides comprehensive data on emissions associated with specific processes and their respective sources. Additionally, it offers valuable insights into the consumption of electricity associated with direct use or process heat production.
(4) A key enhancement to the database is the categorization of energy consumption across four distinct temperature ranges for industrial processes, along with the differentiation of electricity usage. The temperature ranges are defined as: low temperature (<100 °C), medium temperature (100 °C-500 °C), high temperature (500 °C-1000 °C), and very high temperature (>1000 °C). This classification allows for a detailed mapping of process heat requirements across different industrial applications. Additionally, the database distinguishes between electrical energy used directly for machinery operations (such as motors, pumps, and mechanical processes) and that used for generating process heat [13,14].
  To facilitate the further expansion of the database, it was necessary to collate a diverse range of information pertaining to industrial processes across various sectors. This entailed an understanding of the specific energy sources employed, the processes in which they are utilized and the temperatures at which they operate. However, the existing literature on this subject is limited and often lacks sufficient detail, necessitating interpretation and some simplifications to define this aspect of the database. To illustrate, with respect to the cement industry, Sahoo et al. [15] assert that the consumption of electricity is predominantly associated with the crushing and grinding processes, which are mechanical procedures. In contrast, fossil fuels are utilized in the calcination process, which occurs at temperatures ranging from 900 °C. As an additional case in point, in the paper industry, Gambini et al. [16] posit that electricity is primarily employed to operate machinery, whereas the requirement for steam at a temperature of 170 °C is fulfilled by a thermal power plant and a gas boiler.
  All information regarding other industries was derived from: iron and steel industry from Kim et al. [17], food and beverages industry from Sovacool at al. [18], textile industries from Farhana et al. [19], wood products industry from Quesada-Pineda et al. [20], refinery sector from Oliveira et al. [21].
  This work synthesizes the collected information about temperature requirements and electricity usage patterns across different industrial sectors into Fig. 4, which presents the distribution of energy consumption for each industrial sector categorized by process heat temperature ranges (<100 °C, 100 °C-500 °C, 500 °C-1000 °C, >1000 °C) and distinguishes between direct electricity use for machinery and thermal applications.

**Fig. 4:**
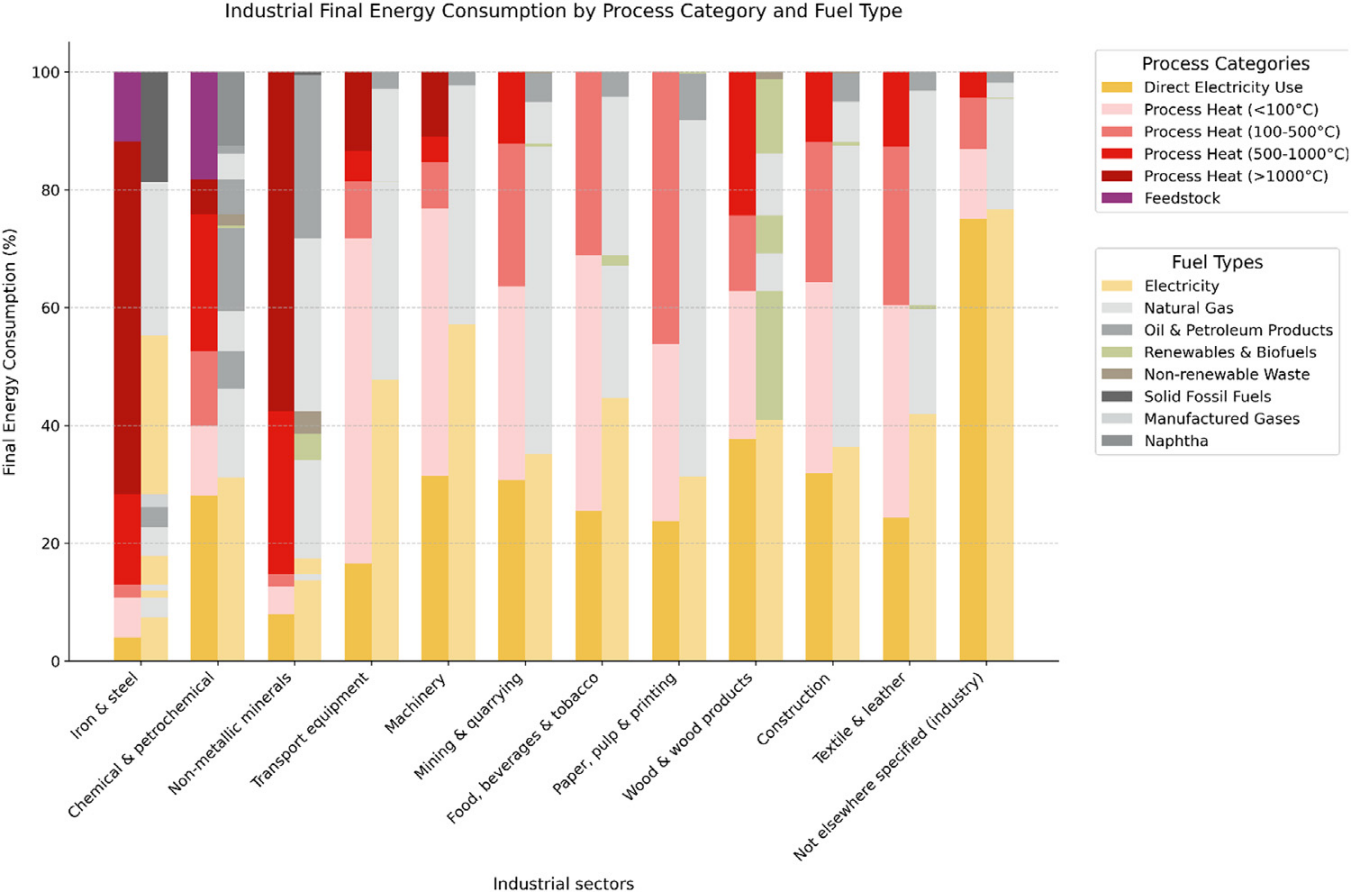
Industrial energy consumption by industrial sector, process category and fuel type.

The final step of the methodology involves combining the two share matrices - the emission mix (Table 2) and the process heat temperature distribution matrix (matrix showed in Fig. 4) - with the site-level emissions data from EU-ETS. This integration allows for a comprehensive mapping of each industrial site's energy consumption pattern. For each facility in the EU-ETS database, the total emissions are first broken down into different energy sources and feedstock uses according to the sector-specific shares from Table 2. These energy source-specific emissions are then converted back to energy consumption using the same emission factors applied earlier. Subsequently, the thermal energy consumption is distributed across the four temperature ranges and direct electricity use according to the sector-specific patterns established in Fig. 2. The result is a detailed database that provides, for each industrial site, a complete breakdown of energy consumption by source (including feedstock) and process heat temperature range, along with associated emissions. This granular information enables precise analysis of decarbonization opportunities and technology options for each facility.

## Limitations

This study represents a significant step forward in the classification of industrial emissions and energy consumption within the Italian context. However, several conceptual and methodological aspects remain open to improvement, which can be considered as limitations. Firstly, it was not feasible to include the total number of industrial sites across Italy. Consequently, the study focuses exclusively on the most energy-intensive facilities, identified from the EU-ETS database based on specific criteria outlined in the Emission Trading Scheme. The industrial sites analyzed in this study account for approximately 80 % of the total emissions in the sector. Moreover, a significant limitation concerns the categorization of energy consumption across distinct temperature ranges, primarily due to the lack of standardized and precise data on the fuels used in various industrial processes and the corresponding process temperatures. As a result, several assumptions were necessary. The available literature offers only limited and often ambiguous information, requiring careful interpretation and refinement to ensure consistency. To address this challenge, a single source was chosen for each industrial sector, serving as the basis for the assumptions made in this study.

## Ethics Statement

The authors confirm that they have read and followed the ethical requirements for publication in Data in Brief. The current work does not involve human subjects, animal experiments, or any data collected from social media platforms. The data presented in this paper is derived from publicly available databases and statistical sources, specifically the European Union Emissions Trading System (EU-ETS), EUROSTAT Energy Balance statistics, and JRC-IDEES database. All data processing and analysis were conducted in accordance with academic standards and ethical research practices.

## CRediT Author Statement

**Enrico Bernelli Zazzera:** Software, Validation, Formal analysis, Methodology, Data Curation, Writing - Original Draft, Writing - Review & Editing, Visualization. **Matteo Giacomo Prina:** Conceptualization, Methodology, Writing - Original Draft, Writing - Review & Editing, Visualization, Supervision. **Riccardo Marchetti:** Writing - Review & Editing. **Steffi Misconel:** Writing - Original Draft, Writing - Review & Editing. **Giampaolo Manzolini:** Writing - Review & Editing, Supervision. **Wolfram Sparber:** Writing - Review & Editing, Supervision, Funding acquisition.

## Declaration of Generative AI and AI-assisted Technologies in the Writing Process

During the preparation of this work the authors used Claude by Antropic in order to improve language and readability. After using this tool/service, the authors reviewed and edited the content as needed and take full responsibility for the content of the publication.

## Data Availability

ZenodoItalian Industrial Site-Level Energy and Emissions Database 2022: A Top-down Disaggregation Methodology (Original data).

## References

[1] European Union Emissions Trading System (EU ETS) data from EUTL, Apr 2024 n.d. https://sdi.eea.europa.eu/catalogue/srv/eng/catalog.search#/metadata/385c4dc1-2c58-430a-9b9a-affc5b358101 (accessed 8 November 2024).

[2] Hotmaps /industrial_sites / industrial_sites_Industrial_Database · GitLab n.d. https://gitlab.com/hotmaps/industrial_sites/industrial_sites_Industrial_Database (accessed 14 November 2024).

[3] Consumo energia elettrica per industria | Terna spa - Terna spa n.d. https://www.collaudo.terna.it/it/sistema-elettrico/statistiche/evoluzione-mercato-elettrico/consumi-settore-industria (accessed 14 November 2024).

[4] Additional data - Eurostat n.d. https://ec.europa.eu/eurostat/web/energy/database/additional-data#Energy%20balances (accessed 14 November 2024).

[5] Statistics | Eurostat n.d. https://ec.europa.eu/eurostat/databrowser/view/nrg_ind_difec/default/table?lang=en&category=nrg.nrg_quant.nrg_quanta.nrg_ind (accessed 14 November 2024).

[6] Joint Research Centre Data Catalogue - JRC-IDEES-2021 - European Commission n.d. https://data.jrc.ec.europa.eu/dataset/82322924-506a-4c9a-8532-2bdd30d69bf5 (accessed 12 December 2024).

[7] Directive (EU) 2023/959 of the European Parliament n.d. https://eur-lex.europa.eu/eli/dir/2023/959 (accessed 22 November 2024).

[8] Codice ATECO • sezione attivitÀ manifatturiere n.d. https://codiceateco.it/sezione?q=C (accessed 14 November 2024).

[9] Database dei comuni italiani gratuito, completo e sempre aggiornato - Garda Informatica n.d. https://www.gardainformatica.it/database-comuni-italiani#scarica (accessed 14 November 2024).

[10] J. Bastos, F. Monforti-Ferrario, G. Melica, GHG emission factors for electricity consumption n.d.

[11] Covenant of Mayors for Climate and Energy - Publications Office of the EU n.d. https://op.europa.eu/en/publication-detail/-/publication/278ae66b-809b-11e7-b5c6-01aa75ed71a1/language-en (accessed 14 November 2024).

[12] Emission factor: naphtha | energy | fuel | global | climatiq n.d. https://www.climatiq.io/data/emission-factor/b0e5ba0b-f4a1-4dca-8f43-a0de33a7df7a (accessed 14 November 2024).

[13] Rehfeldt M., Fleiter T., Toro F. (2018). A bottom-up estimation of the heating and cooling demand in European industry. Energy Effic..

[14] Heat and cooling demand and market perspective - European Commission n.d. https://setis.ec.europa.eu/heat-and-cooling-demand-and-market-perspective_en (accessed November 25, 2024).

[15] Sahoo N., Kumar A., Samsher (2022). Review on energy conservation and emission reduction approaches for cement industry. Environ. Dev..

[16] Gambini M., Vellini M., Stilo T., Manno M., Bellocchi S. (2019). High-efficiency cogeneration systems: the case of the paper industry in Italy. Energies.

[17] Kim J., Sovacool B.K., Bazilian M., Griffiths S., Lee J., Yang M. (2022). Decarbonizing the iron and steel industry: a systematic review of sociotechnical systems, technological innovations, and policy options. Energy Res. Soc. Sci..

[18] Sovacool B.K., Bazilian M., Griffiths S., Kim J., Foley A., Rooney D. (2021). Decarbonizing the food and beverages industry: a critical and systematic review of developments, sociotechnical systems and policy options. Renew. Sustain. Energy Rev..

[19] Farhana K., Kadirgama K., Mahamude A.S.F., Mica M.T. (2022). Energy consumption, environmental impact, and implementation of renewable energy resources in global textile industries: an overview towards circularity and sustainability. Mater. Circ. Econ..

[20] Quesada-Pineda H., Wiedenbeck J., Bond B. (2016). Analysis of electricity consumption: a study in the wood products industry. Energy Effic..

[21] Decarbonisation options for the Dutch refinery sector n.d. https://www.pbl.nl/sites/default/files/downloads/pbl-2020-decarbonisation-options-for-the-dutch-refinery-sector-3659_0.pdf (accessed 22 November 2024).

